# Towards Tailored Patient's Management Approach: Integrating the Modified 2010 ACR Criteria for Fibromyalgia in Multidimensional Patient Reported Outcome Measures Questionnaire

**DOI:** 10.1155/2016/5371682

**Published:** 2016-04-13

**Authors:** Yasser El Miedany, Maha El Gaafary, Sally Youssef, Ihab Ahmed

**Affiliations:** ^1^Rheumatology, Darent Valley Hospital, Dartford DA2 8DA, UK; ^2^Rheumatology and Rehabilitation, Ain Shams University, Cairo, Egypt; ^3^Community, Environmental and Occupational Medicine, Ain Shams University, Cairo, Egypt; ^4^Internal Medicine, Cairo University, Cairo, Egypt

## Abstract

*Objectives*. To assess the validity, reliability, and responsiveness to change of a patient self-reported questionnaire combining the Widespread Pain Index and the Symptom Severity Score as well as construct outcome measures and comorbidities assessment in fibromyalgia patients.* Methods*. The PROMs-FM was conceptualized based on frameworks used by the WHO Quality of Life tool and the PROMIS. Initially, cognitive interviews were conducted to identify item pool of questions. Item selection and reduction were achieved based on patients as well as an interdisciplinary group of specialists. Rasch and internal consistency reliability analyses were implemented. The questionnaire included the modified ACR criteria main items (Symptom Severity Score and Widespread Pain Index), in addition to assessment of functional disability, quality of life (QoL), review of the systems, and comorbidities. Every patient completed HAQ and EQ-5D questionnaires.* Results*. A total of 146 fibromyalgia patients completed the questionnaire. The PROMs-FM questionnaire was reliable as demonstrated by a high standardized alpha (0.886–0.982). Content construct assessment of the functional disability and QoL revealed significant correlation (*p* < 0.01) with both HAQ and EQ-5D. Changes in functional disability and QoL showed significant (*p* < 0.01) variation with diseases activity status in response to therapy. There was higher prevalence of autonomic symptoms, CVS risk, sexual dysfunction, and falling. *Conclusions*. The developed PROMs-FM questionnaire is a reliable and valid instrument for assessment of fibromyalgia patients. A phased treatment regimen depending on the severity of FMS as well as preferences and comorbidities of the patient is the best approach to tailored patient management.

## 1. Introduction

The introduction of the American College of Rheumatology (ACR) 2010 fibromyalgia criteria paved the way for a new era of patient centred approach and better understanding of the disease nature [1]. The concerns raised with the 1990 criteria [2] gave the clues for the development of a new tool based mainly on the patients' perception. However, the ACR 2010 criteria not only changed the fibromyalgia diagnostic approach and case definition, to a disease characterized by self-reported symptoms as well as painful areas, but also provided a broad based severity scale able to stratify the patients according to the level of their fibromyalgia symptoms. A notable attainment of the 2010 criteria was the consequent finding of the Polysymptomatic Distress Scale. In 2011, the diagnostic criteria were revised to include 19 specific pain locations and 6 self-administered symptoms questionnaires including sleep difficulty, fatigue, headache, depression, abdominal pain, and poor cognitive status [3]. This scale consists of the summation of the 2 components of the 2010 criteria, the Widespread Pain Index (WPI) and the Symptom Severity Score (SSS). It was developed in view of the suggestion that “fibromyalgia is a syndrome that lie(s) at the extreme end of the spectrum of polysymptomatic distress.” The Polysymptomatic Distress Scale made it possible to assess not only whether the patient meets the criteria, but also where the patient is on the distress continuum [4, 5].

The prevalence of FMS is remarkably high; it affects 2–5% of the general population, mainly women, the men to women ratio being 1 to 9; those mostly affected were in their forties, although cases among teenagers are increasing [6–9]. However, on using the 2010 classification, with a higher weight of somatic symptoms, the prevalence of FMS was reported to be, even, higher particularly in men [10]. Not surprisingly, FMS is a costly condition, with an estimated cost of 10,000 euros per patient per year [11, 12]

Whilst many associations have been reported with fibromyalgia, including rheumatologic (such as osteoarthritis, Crohn's disease, rheumatoid arthritis, inflammatory bowel disease, systemic lupus, psoriatic arthritis, and Behcet's disease) [13, 14]; medical (such as hepatitis C virus infection, thyroid disease, and HIV) [15, 16]; and psychological (such as major depressive disorder, anxiety, and eating and bipolar disorders) [17–19], less attention was paid to other presenting symptoms and functional disorders such as autonomic dysfunction, falling over, sexual dysfunction, and irritable bowel syndrome. As with other fibromyalgia symptoms included in the new 2010 ACR criteria, these comorbidities should also be assessed and explained to the patients.

This study was carried out aiming at the assessment of the validity, reliability, and responsiveness to change of a patient self-reported questionnaire for fibromyalgia (PROMs-FM) combining the Widespread Pain Index and the Symptom Severity Score as well as construct outcome measures and comorbidities assessment in fibromyalgia patients.

## 2. Patients and Methods

*Patients*. 146 patients presenting with fibromyalgia according to both ACR 2010 and ACR 1999 criteria were consecutively recruited to participate in this study. The PROMs questionnaire was conceptualized based on frameworks used by the WHO Quality of Life tool and the PRO measurement information system (PROMIS). The fibromyalgia symptoms should have been present at a similar level for at least 3 months. The patients who had a disorder that would otherwise explain the pain were excluded from the study. Local ethical and methodological protocols for approval of the study were followed. All patients who participated in the study signed an informed consent according to the Declaration of Helsinki.

### 2.1. Step I: Development of the Fibromyalgia-Specific Item Pool

Initially, cognitive interviews were conducted with 51 fibromyalgia patients (23 males, 28 females; mean age 49.2 ± 8.72 years, mean disease duration 2.1 ± 2.43) diagnosed according to both modified ACR criteria 2010 and ACR 1999 criteria, with a range of severity to identify item pool of questions. Data were recorded using a structured proforma sheet. Interviews took place in a private room and lasted between 30 and 60 minutes. The patients were given the opportunity to identify areas of their lives that were important from their point of view. Item selection and reduction were achieved based on patients as well as an interdisciplinary group of physicians, nurses, and health educators, in addition to clinometric and psychometric methods. The latter included Rasch and internal consistency reliability analyses. Following a content analysis of the transcripts reflecting important patient reported outcomes, the fibromyalgia-specific measures of impairment and health related quality of life were listed. Related themes were highlighted, grouped together, and organised by conceptual categories [20–22]. The content analysis and category identification were discussed between members of the development team and assessed for repetition and ambiguity.

### 2.2. Step II: Development of the Questionnaire

57 registered patients (27 males, 30 females, mean age 39.4 ± 8.9 years, and mean disease duration 2.6 ± 2.5 years) who meet the modified ACR 2010 criteria for fibromyalgia were included in this step of the work. Patients' age, sex, educational level, current marital status, medical history, and work status were collected for each patient included in this study. All participants completed test questionnaires which included the 82 items to be tested. These were given to the patients whilst attending the outpatient clinic in addition to brief introduction letter. A trained nurse was available to help when required. The patients' comments and feedback were recorded by the nurse. Eight patients needed help as they did not have their reading glasses or were unable to read the questionnaire. The goal was to obtain a reliable, statistically valid, unidimensional scale that captured as much as possible (1) the disability continuum and (2) quality of life affection. Using Rasch analysis [23], the items that best balanced and met the criteria of item fit and scale length and were evenly spaced to assess functional impairment were selected for the functional disability assessment (10 items). Similarly the best items to assess quality of life were selected (10 items). For each question in both developed questionnaires (both functional disability and quality of life) there were 4 choices: without any difficulty (=0), with some difficulty (=1), with much difficulty (=2), and unable to do (=3). The score of each questionnaire was the sum of individual item scores divided by 10 or the mean of the item score if 8 or 9 items were completed.

Neither functional disability nor quality of life was scored if fewer than 8 items were completed. Total score for each questionnaire ranges from 0 to 3. Thirty items for fibromyalgia associated comorbidities/systemic affection were also identified.

### 2.3. The Multidimensional Patient Reported Outcome Measures (PROMs) Questionnaire

The questionnaire (Appendix 1) included the following.

(1) The combined health related quality of life questionnaire included 10-item scale to assess functional disability and 10 items to assess quality of life (QoL). The patient should respond using one of the 4 standard response options: 0 = without any difficulty, 1 = with some difficulty, 2 = with much difficulty, and 3 = unable to do. The mean score for each of the functional disability and quality of life indices is calculated and the total score ranged from 0 to 3.

(2) Modified rheumatology attitude index was used to assess self-helplessness including 10-item questions and using numeric rating “0–10 cm” visual analogue scale to score each item. A mean score is calculated across all items. The total score ranged from 0 to 10.

The questionnaires to assess functional disability and quality of life and the modified rheumatology attitude index questionnaires were all developed based on the Rasch model for ordered response options [23].

(3) Disease activity parameters, namely, waking up unrefreshed, fatigue, and trouble thinking or remembering, in addition to levels of affection of mood, pain, sleep, and patient global assessment, were assessed using numeric rating “0–10-cm” horizontal visual analogue scales (VAS) that contain half units, where a score of 0 = no symptoms and a score of 10 = very severe symptoms. The range is 0–10. The 3 parameters, waking up unrefreshed, fatigue, and trouble thinking or remembering, were also highlighted as mild, moderate, and severe.

(4) Assessment of self-reported tender areas was carried out using a table showing all points identified in the revised criteria on both upper and lower limbs. The total number of places where the patient has had pain in the last week was calculated and a score was given out of 19.

(5) Self-reported joint tenderness was carried out on a joint diagram with the joint names written beside it as a guide and the patient was asked to tick the box matching the painful joint(s) [24].

(6) A checklist of 31 somatic symptoms, identified according to the revised fibromyalgia guidelines, was included.

(7) A checklist of 30 fibromyalgia associated comorbidities/systemic affection, including structured “review of systems” (18 questions), 5 questions to assess the falls risk, and 8 questions to assess the cardiovascular risk [25], was included.

In addition, each patient completed a copy of the Stanford HAQ [26] as well as European quality of life questionnaire-5D [27]. The EQ-5D includes single-item measures of mobility, self-care, usual activities, pain/discomfort, and anxiety/depression. Each item is coded using 3 levels (1 = no problems; 2 = some problems; and 3 = severe problems). The instrument includes a global rating of current health using a visual analogue scale (VAS) ranging from 0 (worst imaginable) to 100 (best imaginable). An additional single-item measure of health change (better, much the same, and worse) was included.

### 2.4. Validation

The routine clinic was used as a setting for the questionnaire evaluation. 146 registered patients who meet the modified ACR 2010 criteria for fibromyalgia were included in this step of the work. All patients were asked to complete the PROMs questionnaire while sitting in the waiting area before being examined by the treating physician. A supervising nurse was present to provide help, if needed. The PROMs questionnaire was validated by comparing its yield to a group of other instruments' results that explore different disease activity parameters as well as associated comorbidities including cardiovascular and falls risk assessment.

*Disease Severity Assessment*. This was carried out by the following.

1. Symptom Severity (SS) Score (0–9) was calculated to indicate the severity of the symptoms: fatigue, waking up unrefreshed, and cognitive symptoms, during the past week using the following scale:
2. 0 = no problem; 1 = slight or mild problems, generally mild or intermittent; 2 = moderate, considerable problems, often present and/or at a moderate level; and 3 = severe: pervasive, continuous, life-disturbing problems. A total score out of 9 was given.
3. Somatic symptoms score (0–3) was scored following the scale: 0 = no problem; 1 = few symptoms, slight or mild problems, generally mild or intermittent; 2 = moderate number of symptoms, considerable problems, often present and/or at a moderate level; and 3 = severe, great deal of symptoms which are pervasive, continuous, life-disturbing problems. A total score out of 3 was given.
4. Total Symptom Severity Scale Score (parts 1 + 2) was also calculated ranging from 0 to 12.
5. Total number of places patient has had pain in the last week was assessed in the range of 0–19.
6. The patient's global health assessment (PGH) of disease activity was measured on a continuous 0–10 cm visual analogue scale (VAS).
7. Cardiovascular risk was assessed by SCORE [27].
8. Falls risk was assessed by falls risk assessment questionnaire (FRAS) [28].

### 2.5. Clinical Evaluation

Full history, including disease duration, assessment for articular as well as extra-articular manifestations, revision of the current medications, and assessment for possible cardiovascular as well as falls risks were carried out for every patient. Each patient was then subjected to full clinical examination to assess the parameters of disease activity.

Each patient had a blood check for ESR and CRP levels, lipid profile, rheumatoid factor, anti-CCP, ECG, carotid Doppler, and haemoglobin A_1_c (the erythrocyte sedimentation rate (ESR) was measured using Westergren's method and CRP was measured using ELISA technique.).

### 2.6. Reliability and Comprehensibility

Test-retest reliability (reproducibility) was assessed by asking the patients to complete a second copy of the questionnaire 1 week after the initial visit to the rheumatology department when they completed the first copy. If the patient was in need for one of the fast working therapies, for example, acupuncture or local injections, this was scheduled to be carried out on the 7th day after completing their second copy of the questionnaire. “Analysis of properties of the questionnaire” was set as a justification for completing the questionnaire for the second time. After completing the questionnaire for the first time, every patient was asked to rate the questionnaire out of 10 to assess the comprehensibility.

### 2.7. Responsiveness

Responsiveness has been described as the ability of an instrument to measure clinically important change over time with change at present. Sensitivity to change of the PROMs questionnaire was assessed in 146 patients who were treated according to the Canadian guidelines for fibromyalgia treatment [29] as well as a patient education program. Patients completed the questionnaire before and 6 months after treatment. Average percentage changes in disease severity parameters assessed by PROMs-FM were assessed.

### 2.8. Statistical Analysis

Data were collected regularly and statistical manipulation was performed using the 11th version of SPSS. Variables are summarized in the form of mean and standard deviation if continuous and frequency distribution if categorical. Median and interquartile range (IQR) were calculated for skewed data. Pearson correlation coefficient was used to figure out correlation between quantitative variables. Error bars and scatter diagram were used to illustrate deviations and correlation, respectively, of different variables. Changes in the PROMs questionnaire were calculated by subtracting the second record from the first record. Intraclass correlation coefficient for agreement (reliability) and consistency was calculated, and alpha statistic was calculated as an additional measure of reliability. Validation was tested by calculation of Spearman's correlation coefficient with the tested questionnaire and the selected confirmatory tests. *p* value is significant if less than 0.05.

## 3. Results

One hundred and forty-six patients (71 males, 75 females; mean age 41.6 ± 7.8 years, mean disease duration 2.3 ± 1.9 years) who meet the modified ACR 2010 criteria for fibromyalgia were included in this work to assess the validity and reliability of the PROMs-FM questionnaire.  Table 1 shows clinical and laboratory demographics of the studied group of patients.

*Applicability and Feasibility of the PROMs*. The mean time to complete the questionnaire was 8.46 ± 0.25 minutes. The mean time to scan and score the patient answers was 1.02 + 1.38 minutes, whereas the mean time to record the patient data was 1.08 + 2.61 minutes. One hundred and 34 (91.8%) assigned the PROMs questionnaire as comprehensive giving scores higher than or equal to 8.5. Only 12 patients recorded a score of 7 out of 10. A mean score of 9.4 was reported by the interviewed patients (95% CI 9.2–9.6).

*Validity*. To assess the validity of the PROMs-FM questionnaire items (Appendix  1) were compared to the parameters of disease severity; Table 2 shows correlation of the PROMs-FM items with the disease severity parameters as well as the inflammatory markers (ESR and CRP) in the fibromyalgia patients included in this work.

**Figure 1:**
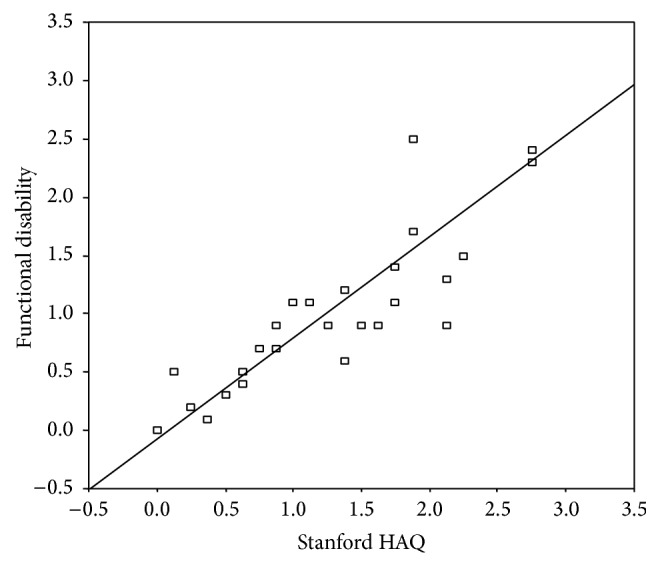
Scatter diagram displaying correlation of the functional disability score with the Stanford HAQ among rheumatoid arthritis patients. *r* = 0.933, *p* value < 0.001.

Comparing the PROMs-FM/functional disability to the Stanford HAQ among patients with fibromyalgia (Figure 1) revealed a significant correlation with *r* = 0.933, *p* < 0.001. Similarly, there was a significant correlation between PROMs-FM/quality of life and EQ-5D score with *r* = 0.882 (*p* < 0.001) as well as EQ-5D VAS score with *r* = 0.891 (*p* < 0.001). There was also a significant correlation between widespread pain score and other parameters of disease severity (*p* < 0.001), whereas there was significant difference on comparing patient reported joint tenderness to the physician reported joint tenderness (*p* < 0.01). There was no correlation between the PROMs-FM recorded outcome measures and the disease severity symptoms and inflammatory markers (namely, ESR and CRP).

*Reliability*. Minimal changes ranging between −0.03 and 0.06 were noticed when repeating the PROMs-FM for functional impairment assessment while the quality of life score demonstrated changes ranging between 0.02 and 0.11 (Table 3). Standardized alpha as well as intraclass correlation coefficient (ICC) showed a relatively high value for the functional impairment, quality of life, and modified rheumatology attitude index scores.

*Responsiveness*. On studying the correlation of percentage changes in PROMs-FM/functional disability and quality of life (QoL), modified rheumatology attitude index, and percentage of change of disease severity parameters, a statistically significant correlation (*p* < 0.001) was observed between percentage of changes of disease severity parameters as assessed with PROMs-FM questionnaire on one side and Widespread Pain Index and total Symptom Severity Score on the other side.  Table 4 shows that the average percentage of change was almost in the same range for the different instruments.

Compared with those unable to work due to ill health, working patients had significantly better levels of functional ability and quality of life (*p* < 0.001).

Assessment of falls risk revealed that 79/146 (54.1%) of the fibromyalgia patients had a positive history of changing their gait or walking speed, whereas 59/146 (40.4%) gave history of more than one fall the past year. Increased falls risk or loss of balance among fibromyalgia patients was significantly correlated (*p* < 0.01) to total Symptom Severity Score, Widespread Pain Index score, HAQ score, and PROMs-FM/functional disability. There was higher prevalence of autonomic symptoms in fibromyalgia patients of 86/146 (58.9%) including cold hands flatulence and tiredness. Similarly, there was increased prevalence of the cardiovascular risk factors among fibromyalgia patients. The prevalence for cardiovascular risk factors among fibromyalgia patients was 38% for hypertension (mean systolic 142.4 mmHg (±13.6), mean diastolic 89.7 mmHg (±14.0)), 21% for diabetes mellitus, 14% for hyperlipidemia, and 12% for ischemic heart disease. The 10-year CV risk among RA patients was 10.9% (±0.64), whereas the 10-year CV risk in fibromyalgia patients was 9.8% (±0.57). 57.5% of the patients (84/146) reported sexual difficulties with their partner (77/146 (52.7%) were males and 66 (47.3%) were females). Among the male patients 25/146 patients (32.5%) put their difficulties down to their joint pain, whereas 67.5% (52/77 patients) attributed it to erectile dysfunction. Sexual dysfunction in both men and women was significantly correlated to severity of depression and mood scores as well as Widespread Pain Index (*p* < 0.01) whereas in men it was also significantly related to increased cardiovascular risk (*p* < 0.01).

## 4. Discussion

Validation is the process by which any data collection instrument, including questionnaires, is assessed for its dependability or in another way the degree to which a questionnaire reflects reality. Construct validity is the ability of a measure to assess correctly a particular cause and effect relationship between the measure and some other factors [30]. Results of this work revealed that the content construct of the PROMs-FM scales for functional disability and quality of life revealed correlation with both HAQ (*r* = 0.93) and EuroQoL-5D scores (*r* = 0.88). Earlier studies carried out on other fibromyalgia questionnaires revealed that the revised version of the Fibromyalgia Impact Questionnaire (FIQR) showed strong correlation with the original Fibromyalgia Impact Questionnaire (FIQ) (*r* = 0.88  *p* < 0.001) and SF-36's physical function and pain subscales (*r* = −0.80 and *r* = −0.60, resp.); the validity of the original version of the FIQ was not tested [31]. Total Combined Index of Severity of Fibromyalgia (ICAF) score shows a moderate correlation with the FIQ (*r* = 0.69) and HAQ (*r* = 0.59), while testing the construct validity of the FFS (fibrofatigue scale) revealed that the correlations of the FFS items with pain score and the physical function subscale of the SF-36 ranged from 0.28 to 0.32 [32]. As far as the global VASFIQ, its score was highly correlated with FIQ scores at baseline (*r* = 0.94). Changes in global VASFIQ and FIQ scores correlate similarly to a patients' global impression of change scale (*r* = 0.58) [33].

Reliability represents the degree to which a questionnaire will produce the same result if administered again or the “test-retest” concept. It is also a measure of the degree to which a questionnaire can reflect a true change. Results of this work revealed good internal consistency of the developed tool (*α* = 0.93). The revised version of the FIQ showed the internal consistency of *α* = 0.95 whereas the FFS questionnaire was *α* = 0.92. This high internal consistency may reflect some overlapping of items or domains. The ICAF showed an overall value of *α* = 0.82 for all items. Similarly the fibromyalgia bladder index (FBI) internal consistency ranged from *α* = 0.76 (for bladder urgency, pain, and nocturia) to *α* = 0.81 (ICSI/ICPS) [34]. There are no internal consistency data available for VASFIQ, fibromyalgia survey questionnaire (FSQ), and fibromyalgia health assessment questionnaire (FHAQ) [33].

Test-retest reliability assessment of the developed PROMs-FM questionnaire was adequate. Values for the interclass correlation coefficient ranged from 0.89 to 0.96. There are no data available for FHAQ or FSQ whereas the ICC values for the FBI ranged from 0.73 to 0.84. Though ICC is the standard tool for assessment of both continuous and ordinal scales whereas Kappa is used for nominal scales, reliability of other fibromyalgia questionnaires was tested using other statistical approaches. Reliability of the FIQ was tested by means of Spearman correlation coefficient 0.85, whereas the FIQR assessed it using Pearson's correlation (*r* = 0.83) in its Turkish version and the ICAF [35].

Responsiveness is usually assessed by examining changes in instrument scores for groups of patients whose health is known to have changed. Results of this work revealed that changes in functional disability, quality of life, and self-helplessness scores in the PROMS-FM showed significant variation with disease activity status and response to therapy. Responsiveness was tested only in the FIQ, the FFS, and the ICAF. The approach to measure responsiveness in FIQ was not that strong, as it was assessed in a clinical trial of acupuncture [36]. It showed an area under the curve of 0.77 to discriminate change, with no clear intervention or anticipated change. The FFS was assessed using Student's *t*-test in patients who showed improvement of the Clinical Global Impressions scale in a 24-week trial [37]. In concordance, the ICAF showed also sensitivity to change [33] whereas FSQ showed an area under the curve of 0.65 compared to the Clinical Severity Index [36].

Very few large FM cohort studies exist in which comorbid disease states are systematically evaluated, and there is sparse data on men. Results of this work revealed increased prevalence of comorbidities in fibromyalgia patients including both increased cardiovascular and falls risks, as well as sexual dysfunction. Earlier data reported coprevalence for some of these comorbidities which varied from 10 to 42% for hypertension, 12 to 40% for osteoporosis, and 4 to 23% for diabetes [38, 39]. Another study [40] reported prevalence of sexual function in their fibromyalgia patients at 97% (30/31 female patients with fibromyalgia). This impairment was associated with the degree of depression. The recognition of these comorbidities and their inclusion for the multidisciplinary management of fibromyalgia may contribute to improving quality of life of these patients.

Do we need another PROMs questionnaire for fibromyalgia? Results of this work revealed that this is the first questionnaire developed integrating the new fibromyalgia criteria and patient reported outcome measures. Moreover it addresses the limitations of previously published questionnaire. PROMs-FM multidimensional questionnaire enable the assessment of the patient's levels of pain, fatigue, global assessment, sleep, cognitive aspects, coping, health related quality of life and functional disability, anxiety or depression, and social functioning. In addition it assesses the disease somatic manifestations, cardiac, falls, and comorbidity risks. Though the FIQ was used as the gold standard for several PRO validation studies, studies revealed that it can underestimate the severity of the patient, as items that are not marked are deleted from the calculation; in addition, the FIQ has a gender bias, as it was developed originally in women [41]. The revised FIQ assesses 3 main domains: function, overall impact, and symptoms, whereas it does not include any assessment for sleep, health related quality of life, cognitive aspects, and coping. The fibrofatigue scale was designed to measure the severity of symptoms in fibromyalgia as well as in chronic fatigue syndrome patients; however, the scale requires a trained administrator, making it potentially unsuitable for large-scale studies or clinical practice [42]. The Combined Index of Severity of Fibromyalgia (ICAF) is a self-administered questionnaire that evaluates FMS main symptoms through five domains: emotional (anxiety and depression), physical (pain, fatigue, sleep quality, and functional ability), active coping (positive coping strategies), passive coping, and global [43]. It has 59 items and the score ranges from 0 to 89; its psychometric properties are very good; however, due to its length and complex scoring system and the fact that it is only available in Spanish, the ICAF has been used seldom. It is a 7-item scale modified VAS of the FIQR that quantifies the severity of FMS. It enables rapid patient assessment and informed treatment decisions in busy clinics. It is widely used in clinical practice and research and has no floor or ceiling effect. However, its psychometric properties are far from excellent and it needs initial training. The VASFIQ should not be used in isolate to make treatment decisions [33].

In conclusion, fibromyalgia is a complex syndrome which represents a challenge to its diagnosis as well as assessment in standard clinical practice. The PROMs-FM is a specific multidimensional patient reported outcome measures questionnaire which is reliable and valid tool for assessment of patients suffering from fibromyalgia. Being short, rapid, and comprehensive, this adds more to its applicability in standard clinical practice. The data support the value of completion of the simple 2-page patient questionnaire, which provides a quantitative written documented record by the patient, at each visit to the clinic. A phased treatment regimen depending on the severity of FMS as well as preferences and comorbidities of the patient is the best approach to tailored patient management.

## Supplementary Material

Patient reported outcome Measures questionnaire (PROMs) for fibromyalgia patients.

## Figures and Tables

**Table 1:** Demographic and clinical characteristics of the studied patients.

Characteristic	Parameter
Age, mean (SD)	41.6 (7.8)
Female, *N* (%)	75 (51.4%)
Disease duration in years, mean (SD)	2.3 (1.9)
Tender point count by patient (WPI), mean (SD)	14.1 (3.8)
Tender point count by physician, mean (SD)	13.1 (2.7)
Tender joint count by patient, mean (SD)	7.2 (3.6)
Tender joint count by physician, mean (SD)	2.2 (2.4)
HAQ mean (SD)	2.73 (0.7)
PROMs-FM functional disability	2.72 (0.6)
PROMs-FM quality of life	2.93 (0.9)
EQ-5D (TTO score)	−0.349
EQ-5D (VAS 0–100)	0.038
Modified rheumatology attitude index	8.8 (1.7)
ESR, mean (SD) mm/h	22.2 (16.3)
CRP, mean (SD) mg/dL	9.6 (2.6)

WPI: Widespread Pain Index; ESR: erythrocyte sedimentation rate, and CRP: C-reactive protein.

**Table 2:** Fibromyalgia patients: correlation of the PROMs items with the disease activity parameters as well as the inflammatory markers (ESR and CRP) as validating tools.

Items of the PROMsQ	WPI	Somatic symptoms	Sleep disturbance	EQ-5D	HAQ	ESR	CRP	Total severity score
PROMs-FM/Fn. Dis.	0.642^*∗∗*^	0.668^*∗*^	−0.661^*∗∗*^	0.619^*∗∗*^	0.933^*∗∗*^	0.219	0.169	0.641^*∗∗*^
PROMs-FM/QoL	0.793^*∗∗*^	−0.689^*∗∗*^	−0.780^*∗∗*^	0.882^*∗∗*^	0.552^*∗∗*^	0.621	0.221	0.782^*∗∗*^
Pain score	0.856^*∗∗*^	0.763^*∗∗*^	−0.869^*∗*^	0.678^*∗∗*^	0.513^*∗∗*^	0.106	0.182	0.645^*∗∗*^
Patient global assessment	0.718^*∗∗*^	0.621^*∗∗*^	−0.756^*∗∗*^	0.546^*∗∗*^	0.250^*∗∗*^	0.121	0.221	0.671^*∗∗*^
Fatigue score	0.828^*∗∗*^	0.625^*∗∗*^	0.728^*∗∗*^	0.664^*∗*^	0.559^*∗∗*^	0.128	0.178	0.764^*∗∗*^
Unrefreshing sleep	0.713^*∗∗*^	0.612^*∗∗*^	0.849^*∗∗*^	0.697^*∗∗*^	0.648^*∗∗*^	0.079	0.279	0.464^*∗∗*^
Trouble thinking	0.822^*∗∗*^	0.716^*∗∗*^	−0.561^*∗∗*^	0.583^*∗∗*^	0.453^*∗∗*^	0.102	0.102	0.576^*∗∗*^
mRAI	0.662^*∗∗*^	0.735^*∗∗*^	−0.672^*∗∗*^	0.596^*∗∗*^	0.684^*∗∗*^	0.059	0.229	0.741^*∗∗*^

^*∗*^ *p* < 0.05.

^*∗∗*^ *p* < 0.01.

WPI: Widespread Pain Index.

HAQ: health assessment questionnaire, EQ-5D: European quality of life questionnaire-5D, mRAI: modified rheumatology attitude index, ESR: erythrocyte sedimentation rate, and CRP: C-reactive protein.

PROMs-FM/Fn. Dis.: patient reported outcome measures questionnaire-fibromyalgia/functional disability.

PROMs-FM/QoL: patient reported outcome measures questionnaire-fibromyalgia/quality of life.

**Table 3:** Reproducibility of PROMs-FM questionnaire.

	First measure Mean (SD)	Change Mean (95% CI)	Standardized alpha	ICC (95% CI)
PROMs-FM/Fn. Dis.	2.7 (0.6)	0.01 (−0.03–0.06)	0.982	0.935 (0.915–0.955)
PROMs-FM/QoL	2.9 (0.9)	0.07 (0.02–0.11)	0.9645	0.931 (0.912–0.947)
WPI	6.4 (1.2)	0.11	0.891	0.83 (0.81–0.85)
Fatigue score	8.6 (1.1)	0.1	0.911	0.85 (0.83–0.87)
Unrefreshing sleep	9.1	0.08	0.886	0.86 (0.84–0.87)
Trouble thinking	8.9	0.09	0.914	0.84 (0.81–0.87)
mRAI	7.6 (0.47)	0.07 (0.05–0.09)	0.942	0.944 (0.936–0.952)

PROMs-FM/Fn. Dis.: patient reported outcome measures questionnaire-fibromyalgia/functional disability.

PROMs-FM/QoL: patient reported outcome measures questionnaire-fibromyalgia/quality of life.

WPI: Widespread Pain Index.

ICC: intraclass coefficient.

**Table 4:** Average percentage changes in disease severity parameters assessed by PROMs-FM.

	Mean	SD	95% CI
PROMs-FM/Fn. Dis.	62.68	30.02	59.96–77.61
PROMs-FM/QoL	60.37	31.30	59.80–78.72
WPI	67.62	33.2	58.41–76.83
Pain score	66.83	34.4	57.72–77.15
Fatigue score	69.74	29.4	59.93–76.81
Unrefreshing sleep	63.62	31.30	59.82–77.06
Trouble thinking	61.58	28.54	57.83–73.51
Total severity score	62.55	27.17	58.53–74.54
Somatic score	65.47	27.71	58.85–75.75

PROMs-FM/Fn. Dis.: patient reported outcome measures questionnaire-fibromyalgia/functional disability.

PROMs-FM/QoL: patient reported outcome measures questionnaire-fibromyalgia/quality of life.

WPI: Widespread Pain Index.
