# Supplementary material for: Towards Tailored Patient's Management Approach: Integrating the Modified 2010 ACR Criteria for Fibromyalgia in Multidimensional Patient Reported Outcome Measures Questionnaire
Source: Arthritis. 2016 Apr 13;2016:5371682. doi: 10.1155/2016/5371682 (PMC4846760; doi:10.1155/2016/5371682)
Supplement: Supplementary file 1 — Patient reported outcome Measures questionnaire (PROMs) for fibromyalgia patients. [file 5371682.f1.docx]

Multi-Dimensional Questionnaire for Patient Reported Outcome Measures - Fibromyalgia

This questionnaire includes information not available from blood tests, X-rays, or any source other than you. Please try to answer each question. There is **no right or wrong answer**. Please answer exactly as **YOU** think or feel.

**1.We are interested in learning how your illness affects your ability to function in daily life. Please tick (√) the ONE best answer that describes your usual abilities OVER THE PAST WEEK:**

**Over the LAST WEEK, were you able to**  Without With With Unable

### Fn. Disability

ANY SOME MUCH TO DO

Difficulty Difficulty Difficulty

**1**. **Get on and off the toilet? ..……… ……….. ……….. ……….**

**2. Dress yourself, including tying shoelaces & putting on socks ..……… ……….. ……….. ……….**

**3. Bend down to pick up object off the floor ..……… ……….. ……….. ……….**

**4. Sit for long periods of time e.g. working on flat topped ..……… ……….. ……….. ……….**

**QoL**

**table or desk**

**5. Lie down / sleep on your back ..……… ……….. ……….. ……….**

**6. Stand up from a chair without arms? ..……… ……….. ……….. ……….**

**7. Walk outdoors on flat ground including crossing the road ..……… ……….. ……….. ……….**

**8. Play with / look after children ..……… ……….. ……….. ……….**

**9. Go up 2 or more flights of stairs ..……… ……….. ……….. ……….**

**10.** **Do outside work (such as DIY/ gardening/ lifting) ..……… ……….. ……….. ……….**

**Not Applicable**

**1. Get a good night’s sleep? ..……… ……….. ……….. ………. ………**

**2. Deal with the usual stresses of daily life? ..……… ……….. ……….. ………. ………**

**3. Cope with social/ family activities? ..……… ……….. ……….. ………. ………**

**4. Deal with feelings of anxiety or being nervous? ..……… ……….. ……….. ………. ………**

**5. Deal with feelings of low self esteem or feeling blue? ..……… ……….. ……….. ………. ………**

**6. Get going in the morning? ..……… ……….. ……….. ………. ………**

**7. Do your work as you used to do? ..……… ……….. ……….. ………. ………**

**8. Deal with any worries about your future? ..……… ……….. ……….. ………. ………**

**9. Continue doing things you used to do, despite tiredness? ..……… ……….. ……….. ………. ………**

**10. Continue your relationship with your partner (husband/wife)?** **..……… ……….. ……….. ………. ………**

**Sleep**

**Unref. sleep**

3**. How much of a problem has waking up UN-REFRESHED been for you OVER THE PAST WEEK?**

### PGA

**2. How much of a problem has SLEEP (i.e., resting**

**at night) been for you OVER THE PAST WEEK?**

5 15 25 35 45 55 65 75 85 95

5 15 25 35 45 55 65 75 85 95

**NO**  **SEVERE NO SEVERE**

**Prob. Prob. Prob. Prob.**

0 10 20 30 40 50 60 70 80 90 100 0 10 20 30 40 50 60 70 80 90 100

🡸 Severe 🡺

🡸 Mod 🡺

🡸 Mild 🡺

**4. Considering all the ways your Symptoms may be affecting you AT THIS TIME**

**Please put a circle around the number that best indicates how well you are doing:**

5 15 25 35 45 55 65 75 85 95

**VERY** **VERY**

**WELL** **POORLY**

0 10 20 30 40 50 60 70 80 90 100

6. How much of a problem has Trouble Thinking or remembering been for you OVER THE PAST WEEK?

### Fatigue

Thinking

5. How much of a problem has UNUSUAL FATIGUE

or tiredness been for you OVER THE PAST WEEK?

5 15 25 35 45 55 65 75 85 95 5 15 25 35 45 55 65 75 85 95

No Fatigue Severe No Prob. Severe

0 10 20 30 40 50 60 70 80 90 100 0 10 20 30 40 50 60 70 80 90 100

🡸 Severe 🡺

🡸 Mod 🡺

🡸 Mild 🡺

🡸 Mild 🡺

🡸 Mod 🡺

🡸 Severe 🡺

### Pain

**Mood**

**8. OVER THE PAST WEEK** **how much of a problem has your mood (feeling down / anxious) affected you?**

**7. OVER THE PAST WEEK how would you**

**rate the severity of your body PAIN?**

**NO SEVERE No Worst**

5 15 25 35 45 55 65 75 85 95

2.5 7.5

**Pain Pain Effect Effect**

0 10 20 30 Mild 40 50 60 70 Mod.80 90 100 0 1 2 3 Mild 4 5 6 7 Mod. 8 9 10

**Please place a (X) in the appropriate box to indicate in which of your JOINTS you feel painful TODAY.**

**Please place (√) in front of the painful area(s) over your body which you feel painful TODAY.**

**Wolfe et al. ACR 2010**

| **Right** | **Left** | | | |
| --- | --- | --- | --- | --- |
| **Upper Limb** | | | | |
| **Shoulder Girdle** | | **Shoulder Girdle** | | |
| **Upper Arm** | | **Upper Arm** | | |
| **Lower Arm** | | **Lower Arm** | | |
| **Lower Limb** | | | | |
| **Outer Hip Area** | | **Outer Hip Area** | | |
| **Upper Leg** | | **Upper Leg** | | |
| **Lower Leg** | | **Lower Leg** | | |
| **Jaw** | | | | |
| **Jaw (Rt.)** | | **Jaw (Lt.)** | | |
| **Trunk** | | | | |
| **Neck** | | **Chest** | | |
| **Upper Back** | | **Abdomen** | | |
| **Lower Back** | | ***WPI score: /19*** | | |
| **_Have you experienced any of these symptoms in the_**  **_past months? “Yes” “No”_** | | | | |
| **_Pain or Cramps on the Lower Abdomen_** | | |  |  |
| Depression | | |  |  |
| Headache | | |  |  |

Shoulder

Elbow

**Hip**

**Knuckles**

**/ Fingers**

**Knee**

**Ankle**

**Top Foot**

Wrist

**Neck**

**Shoulder Blade**

**Rt**

**Lt**

**Toes**

**Low Back**

**Sacroiliac Joint**

**WPI score:**

**(0-19)**

**Somatic Symp.:**

**Tender**

**Joints**

**Please tick () if you have experienced or diagnosed with any of the following OVER THE LAST 6-MONTHs**:

| **Somatic Symptoms** | | | | | |  | **Other Systemic Diseases** | |  | **Cardiovascular Risk Assessment** | | |
| --- | --- | --- | --- | --- | --- | --- | --- | --- | --- | --- | --- | --- |
| **Fever** | |  | | **Dry Eye / Dry Mouth** | |  | **Loss of height / Vertebral Fracture** | |  | Age > 50 years old | |  |
| **Hair loss** | |  | | **Mouth ulcers** | |  | **Osteoporosis** | |  | High Blood pressure | |  |
| **Muscle pain** | |  | | **Easy bruising** | |  | **Recent Fractures** | |  | High Cholesterol | |  |
| **Muscle weakness** | |  | | **Irritable Bowel syndrome** | |  | **Vitamin D deficiency** | |  | **Current Smoker** | |  |
| **Chest Pain** | |  | | **Headache** | |  | **Thyroid Disease** | |  | **Ischemic heart Disease** | |  |
| **Blurred vision** | |  | | **Wheezing in the chest** | |  | **Parathyroid gland Disease** | |  | **Stroke** | |  |
| **Hearing difficulties** | |  | | **Cough/ Shortness of breath** | |  | **Hepatitis C** | |  | **Overweight/under weight** | |  |
| **Itching** | |  | | **Heartburn** | |  | **Diagnosed to have cancer** | |  | **Diabetes Mellitus** | |  |
| **Loss of appetite** | |  | | **Dark or bloody stools** | |  | **Absent from work due to body pains** |  | | **Falls Risk Assessment** | | |
| **Pain/cramps in the abdomen** | |  | | **Feeling Sickly / Nausea** | |  | **Short plans for having a baby** | |  | **>1 Fall in the last year** | |  |
| **Rash** | |  | | **Constipation** | |  | Sexual relationship Problems | |  | **Problems with your sight** | |  |
| **Raynaud’s phenomenon** | |  | | **Diarrhea** | |  | **Problems with erection (for men)** | |  | Loss of your balance | |  |
| **Ringing in ears** | |  | | **Problems with urination** | |  | **Psoriasis** | |  | **Change in Gait / Walking Speed** | |  |
| **seizures** | |  | | **Bladder spasms** | |  | **Coeliac disease** | |  | **Weakness of your grip strength** | |  |
| **Sun Sensitivity** | |  | | **Numbness/tingling** | |  | **Recent viral infection** | |  | For Official Use: Scores (0-3) | | |
| **Taste changes/Loss of taste** | |  | | **Problems with thinking/memory** | |  | **Registered Disabled** | |  | **Fatigue:** | **Unrefresh.:** | |
| **No Symp.(0): □** | **Few Symp.(1):□** | | **Mod. Symp.(2): □** | | **Several Symp.(3): □** | | **Recent viral infection** | |  | **Cog.Symp.:** | **WPI: /19** | |

**The statements below concern your personal beliefs. Please circle the number that best describes**

**how do you feel about the statement. 0 = Not at all; 10 = Strongly Agree**

## mRAI

| **My condition is controlling my life.**      0 1 2 3 4 5 6 7 8 9 10 | **0 1 2 3 4 5 6 7 8 9 10** |
| --- | --- |
| **2. I would feel helpless if I could not rely on other people for help**  **with my condition.**    0 1 2 3 4 5 6 7 8 9 10 |  |
| **3. I am concerned that medicines can not help me.** |  |
|   0 1 2 3 4 5 6 7 8 9 10  **4. I’ve concerns regarding side effects of medications used to treat my condition.**    0 1 2 3 4 5 6 7 8 9 10 |  |
| **5. I often do not take my medicines as directed.**    0 1 2 3 4 5 6 7 8 9 10 |  |
| **6. No matter what I do, or how hard I try, I just can not seem to**  **get relief from my symptoms.**    0 1 2 3 4 5 6 7 8 9 10 |  |
| **7. I am not coping effectively with my condition.**    0 1 2 3 4 5 6 7 8 9 10 |  |
| **8. Sometimes I feel my condition is beyond both my and my doctor’s control.**    0 1 2 3 4 5 6 7 8 9 10 |  |
| **9. Sometimes my condition makes me feel like giving up.**    0 1 2 3 4 5 6 7 8 9 10 |  |
| **10. Due to my condition, sometimes I feel I am a burden to those**  **close to me.** |  |
